# Phylogenetic Relationships among Whiteflies in the *Bemisia tabaci* (Gennadius) Species Complex from Major Cassava Growing Areas in Kenya

**DOI:** 10.3390/insects8010025

**Published:** 2017-02-23

**Authors:** Duke M. Manani, Elijah M. Ateka, Steven R. G. Nyanjom, Laura M. Boykin

**Affiliations:** 1 Department of Biochemistry, Jomo Kenyatta University of Agriculture and Technology, Nairobi P.O. Box 62000-00200, Kenya; snyanjom@gmail.com; 2 Department of Horticulture, Jomo Kenyatta University of Agriculture and Technology, Nairobi P.O. Box 62000-00200, Kenya; eateka@agr.jkuat.ac.ke; 3 Australian Research Council Centre of Excellence in Plant Energy Biology, The University of Western Australia, Crawley, WA 6009, Australia; 4 School of Molecular Sciences, The University of Western Australia, Crawley, WA 6009, Australia

**Keywords:** *Bemisia tabaci*, genetic diversity, mtCOI, cassava, Kenya, cassava brown streak disease (CBSD), cassava mosaic disease (CMD)

## Abstract

Whiteflies, *Bemisia tabaci* (Gennadius) are major insect pests that affect many crops such as cassava, tomato, beans, cotton, cucurbits, potato, sweet potato, and ornamental crops. *Bemisia tabaci* transmits viral diseases, namely cassava mosaic and cassava brown streak diseases, which are the main constraints to cassava production, causing huge losses to many small-scale farmers. The aim of this work was to determine the phylogenetic relationships among *Bemisia tabaci* species in major cassava growing areas of Kenya. Surveys were carried out between 2013 and 2015 in major cassava growing areas (Western, Nyanza, Eastern, and Coast regions), for cassava mosaic disease (CMD) and cassava brown streak disease (CBSD). Mitochondrial cytochrome oxidase I (mtCOI-DNA) was used to determine the genetic diversity of *B. tabaci*. Phylogenetic trees were constructed using Bayesian methods to understand the genetic diversity across the study regions. Phylogenetic analysis revealed two *B. tabaci* species present in Kenya, sub-Saharan Africa 1 and 2 comprising five distinct clades (A–E) with percent sequence similarity ranging from 97.7 % to 99.5%. Clades B, C, D, and E are predominantly distributed in the Western and Nyanza regions of Kenya whereas clade B is dominantly found along the coast, the eastern region, and parts of Nyanza. Our *B. tabaci* clade A groups with sub-Saharan Africa 2-(SSA2) recorded a percent sequence similarity of 99.5%. In this study, we also report the identification of SSA2 after a 15 year absence in Kenya. The SSA2 species associated with CMD has been found in the Western region of Kenya bordering Uganda. More information is needed to determine if these species are differentially involved in the epidemiology of the cassava viruses.

## 1. Introduction

Cassava (*Manihot esculenta* Crantz, Euphorbiaceae) is an important source of food to more than one fifth of the world’s population spread over Africa, Asia, and South America [1]. From the time of its introduction, the crop has spread and gained prominence as a major food and staple crop for many communities in sub-Saharan Africa [2]. On a global basis, cassava is ranked third most important source of carbohydrates in Africa, and it is the second most important food crop after maize in western and coastal regions of Kenya [3]. The cassava crop has a wide range of uses; it is a food security-crop which is estimated to be consumed by approximately 500 million people in Africa [4,5]. Cassava is also a cash crop, livestock feed, and a raw material for industrial uses such as pharmaceutical, starch, and alcohol production [6,7].

Cassava roots are a rich source of carbohydrate while leaves are high in proteins, minerals, and vitamins. The roots of cassava save many lives during famine conditions, especially in various parts of Kenya that experience drought, thereby playing a major role in food security and contributing to poverty reduction [2]. Despite this, the crop is affected by two viral diseases namely cassava mosaic disease (CMD) and cassava brown streak virus disease (CBSD). CMD has been a major biotic constraint to cassava production in Africa [8]. In Kenya, the disease is predominantly caused by Geminiviruses, namely *African cassava mosaic virus* (ACMV) and *East African cassava mosaic virus* (EACMV) [9]. Symptoms of CMD include leaf chlorotic mottle, distortion of leaves, stem twisting, and crinkling and stunting of cassava plant parts [4,8]. The pandemic affected cassava growing areas in East and Central African countries and has caused severe losses in cassava yields which have been estimated to be approximately 50% [7,10,11,12,13]. CBSD, on the other hand, is caused by *Cassava brown streak virus* (CBSV) and *Uganda cassava brown streak virus* (UCBSV) [11,14]. Recent studies have revealed there are several species of CBSD [14], and CBSD has an accelerated rate of evolution [15]. The disease is characterized by severe chlorosis and necrosis on infected leaves, giving them a yellowish, mottled appearance. Chlorosis may be associated with the veins, spanning from the mid vein, secondary and tertiary veins, or rather in blotches unconnected to veins [16]. Brown streaks may appear on the stems of the cassava plant, but in some varieties, a dry brown-black necrotic rot of the cassava root exists, which may progress from a small lesion to the whole root. Finally, the roots may become constricted due to the tuber rot with overall plant stunting, thereby reducing production [17]. The viruses causing CMD and CBSD are transmitted by the whiteflies (*Bemisia tabaci*) and through infected cuttings. Heavy infestation of *B. tabaci* on cassava leads to the presence of honey dew and sooty mould that affects the photosynthetic structures reducing cassava production [4,18].

### 1.1. Whiteflies as Insect Vectors

**Figure 1:**
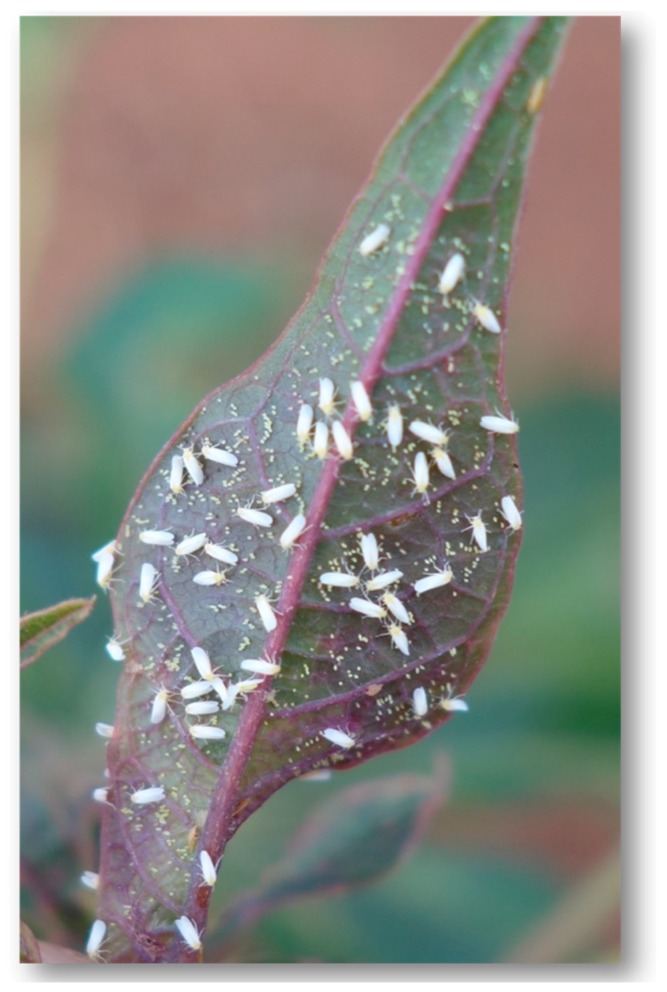
*Bemisia tabaci* SSA1 species on cassava (Photo: Laura M. Boykin).

There are over 1500 whitefly species known worldwide in approximately 126 genera [19]. *Bemisia tabaci* is a species complex that is globally distributed [19] and important because a number of the species that make up the complex are known to damage commercially important plant species either through direct feeding or through the transmission of more than 150 plant viruses primarily belonging to the genus *Begomovirus* (family: Geminiviridae) [20,21,22]. It is, therefore, important to control whiteflies with the aim of reducing virus transmission and agronomic losses. The male adult whitefly is about 0.8 mm while the female is approximately 1 mm in length. Both sexes have wings that are generally opaque and covered with a whitish powder or wax [19]. The whiteflies undergo incomplete metamorphosis. The females lay 50 to 400 eggs underneath the leaves. The whitish eggs range from 0.10 mm to 0.25 mm and change to a brown colour towards the time of hatching within five to seven days. Female whiteflies are diploid and emerge from fertilized eggs whereas male whiteflies are haploid and emerge from unfertilized egg [19]. After the egg stage, the whitefly hatchling develops through four instar stages. In the first instar, commonly called the crawler, the nymph is 0.3 mm in size and grows to be 0.6 mm until the fourth instar stage. During the first instar stage the body is greenish in colour and flat in body structure. The mobile whitefly nymph walks on plants to find a suitable area on the leaf with adequate nutrients and moults into four other instar or nymphal stages over the span of 40–50 days until it reaches adulthood. During moulting, the whitefly nymphs shed their silver skins, which are left on the leaves. At the feeding sites, the nymphs use parts of their mouth to stab into the plant and consume the plant’s cell sap. The next stage is the pupal stage when the eyes become a deep red colour; the body colour becomes yellow, while the body structure thickens. After development is completed, adult whiteflies have light yellow bodies and white wings (Figure 1, [23]).

### 1.2. Bemisia tabaci Species Complex

The *B. tabaci* species complex is globally distributed and the putative species are named based on their geographic locations; Mediterranean; Middle East-Asia Minor 1; Middle East-Asia Minor 2; Indian Ocean; Asia I; Australia/Indonesia; Australia; China; China 2; Asia II 1; Asia II 2; Asia II 3; Asia II 4; Asia II 5; Asia II 6; Asia II 7; Asia II 8; Italy; sub-Saharan Africa 1 (SSA1); sub-Saharan Africa 2 (SSA2); sub-Saharan Africa 3 (SSA3); sub-Saharan Africa 4 (SSA4); sub-Saharan Africa 5 (SSA5); New World; and Uganda [8]. Recent studies have reported new species (Asia II 9, Asia II 10, Asia III, and China 3, and Asia I-India and New World 2), for a total number of 34 morphologically [24] indistinguishable species reported in the *B. tabaci* complex [25,26,27]. The worldwide spread of emerging species, such as *B. tabaci* MEAM1, also known as *B. argentifolii*, and a *Bemisia tabaci* MED, continue to cause severe crop losses which will likely continue to increase, resulting in higher pesticide use on many crops (tomato, beans, cassava, cotton, cucurbits, potato, sweet potato). In East Africa, there are two distinct cassava-associated *B. tabaci* putative species, sub-Saharan Africa 1 (SSA1) and sub-Saharan Africa 2 (SSA2) [28]. In Kenya, limited studies on *B. tabaci* have been carried out in Eastern, Coastal Kenya, Nyanza, and the Western regions of Kenya, and the putative *B. tabaci* species found to be widely spread among the affected crops [29] remain elusive.

### 1.3. Host Range

*Bemisia tabaci* is a polyphagous species, and some species are extremely polyphagous. They colonize mainly annual, herbaceous plants including over 500 species from 74 families [1,30]. *B. tabaci* is known to have a host range that is highly variable. Examples of the wide host range include avocado, banana, cabbage, capsicum, cassava, cauliflower, citrus, coconut, cotton, eggplant, garlic, guava, legumes, mango, mustard, onion, peachy, pepper, radish, squash, soybean, tomato, and tobacco [18]. However, species of *B. tabaci* vary with respect to geography, fecundity, dispersal behaviour, insecticide resistance, natural enemy complex, invasive behaviour, plant virus transmission, and complement endosymbionts [30]. In West Africa and Uganda, differences in host selection has been documented among different *B. tabaci* species [20].

### 1.4. Species Molecular Markers

In molecular genetics analysis, molecular markers have been used to study insect populations and determine phylogenetic relationships. The markers have been used to examine protein-coding genes, major ribosomal RNA genes, and non-coding regions [5,31]. The mitochondrial (mtCOI) DNA marker is the most commonly used, but other markers have also been used—for example, the ribosomal RNAs [32] and a ribosomal nuclear marker of the internal transcribed spacer I (ITSI) region sequences [33]. These markers have the advantage of relative ease of isolation and amplification and are amenable to straightforward analyses. Mitochondrial cytochrome oxidase I (mtCOI) has the highest degree of variability for the *B. tabaci* species compared to the nuclear genes mentioned above, therefore becoming the most widely used marker for phylogenetic studies of *B. tabaci* globally [31,34]; mtCOI has also been sequenced in many different orders of insects. The mitochondrial cytochrome oxidase I (mtCOI) marker [35] and (ITSI) region sequences [33,36] have also been used to study the genetic variability and evolutionary relationships among *B. tabaci* from different geographical locations and host-plant species [26].

## 2. Materials and Methods

### 2.1. Whitefly Collection

The study was conducted in major cassava producing areas of Kenya from 2013 to November 2015. The regions of survey were as follows; Bungoma, Busia, Kakamega, Homabay, Migori, Kisumu, Siaya, Kilifi, Kwale, Taita Taveta, Nyamira, Kitui, and Machakos counties (Table 1). These geographical locations share a similar agro-ecology, where western region counties of Kenya are characterized by bimodal rainfall ranging from 950 to 1500 mm annually, temperature ranging from 18.4 to 25.4 °C, altitude ranges of 900–1800 m, and a savannah grassland. In the Eastern region, the altitude ranges from 1000 m to 1800 m, with a similar rainfall potential of 500–760 mm as the hot and dry coastal hinterland [37]. The Coast region has rainfall ranging from 500 to 1000 mm annually, temperature ranges between 22.4 and 30.3 °C, altitude ranges of 900–1800 m, and a savannah grassland. In each of the regions, 12 fields were randomly selected approximately 10 km apart. In each field, plants were assessed for whitefly populations along X-shaped transects [5]. In all the sites surveyed, adult whiteflies were collected from the five top most cassava leaves from ventral surfaces using an aspirator. Collected adult whiteflies were preserved in 70% ethanol and stored at −20 °C in sampling bottles until analysis in the laboratory [5,38].

### 2.2. DNA Extraction

Individual adult whiteflies preserved in ethanol were selected, washed in distilled water, and dried on filter paper for few seconds. They were then immediately and thoroughly macerated with a micropestle in a 0.5 mL Eppendorf tube containing 50 µL of STE buffer (0.1 M NaCl, 10 mM Tris-HCl, pH 8.0, 1.0 mM EDTA). There was an addition of proteinase K to the STE buffer. The lysis product was incubated for 15 min at a temperature of 65 °C, and then further heat treated at 95 °C for 10 min. The lysis product was centrifuged briefly for 5 min at 10,000 rpm at 4 °C and immediately placed on ice before PCR amplification. Proteinase K was added with a slight modification of this protocol [39].

### 2.3. PCR Amplification of mtCOI DNA

A total of 44 sample sites were collected based on the description above. These collections were used to study the genetic diversity (by extracting DNA from individual whiteflies from each sample collection) and their distribution in the various cassava growing zones in Kenya. Polymerase Chain Reaction was conducted using two primers; MT10/C1-J-2195 (5′-TTGATTTTTTGGTCATCCAGAAGT-3′) and MT12/L2-N-3014 (5′-TCCAATGCACTAAT-CTGCCATATTA-3), to amplify mitochondria cytochrome oxidase I (mtCOI) DNA. All reactions contained 25 µL of 0.15 µL of 60 ng/µL of DNA, 0.5 µL of each primer 10 µm/10 pmole, 0.2 µL of Taq DNA polymerase, 5 µL of 5× MyTaq Reaction buffer, 5.0 µL of DNA template (5xTaq Master enhancer), and topped with 13.80 µL nuclease free water. The contents in the microtube were vortexed briefly and quickly spun. Initial denaturation of template DNA was conducted for 3 min followed by 30 cycles of denaturation at 94 °C for 30 s, primer annealing at 52 °C for 30 s, and extension at 72 °C for 1 min. The final extension of 10 min was run at 72 °C and the reaction held at 4 °C in a Perkin Elmer DNA thermal cycler [35].

### 2.4. Gel Electrophoresis and DNA Sequencing

The PCR products were electrophoresed in 2% Agarose gel stained in ethidium bromide in 1 × Tris acetate ethylenediaminetetraacetic acid (TAE) buffer and the amplified DNA was visualized under UV transilluminator and photographed using an Electrophoresis Documentation and Analysis System 120 digital camera [5]. PCR products of the expected size (850 bp) were obtained. Bands were excised from the agarose gel and purified using a Qiagen gel Purification kit (QIAGEN Inc, San Diego, CA, USA) as per the manufacturer’s procedure [8]. Sequencing was outsourced, and done bi-directionally using the amplification primers. The sequences of COI were submitted to GenBank using Bankit, a web-based data submission tool [40].

### 2.5. Phylogenetic Analysis of mtCOI Sequences

*B. tabaci* mtCOI sequences were edited manually using DNA MAN programme to produce a consensus sequence (~850 bp) for each individual adult whitefly. The edited consensus sequences were aligned using the Clustal W (weighted) [41] MrBayes version 3.2.1 [42] that employs Markov Chain Monte Carlo (MCMC) sampling to approximate the posterior probabilities of phylogenies [43]; the posterior probabilities are shown above the branches (Figure 3). MrBayes 3.2.1 was run in parallel on the Magnus supercomputer (located at Pawsey Supercomputer Centre, Perth, Western Australia) utilising the BEAGLE library [44]. MrBayes 3.2.1 was run with a GTR + I + G model of molecular evolution, utilizing four chains for 30 million generations and trees were sampled every 1000 generations. All runs reached a plateau in likelihood score, which was indicated by the standard deviation of split frequencies (0.0015), and the potential scale reduction factor (PSRF) was close to one, indicating that the MCMC chains converged.

## 3. Results

### Distribution of Bemisia tabaci Putative Species on Cassava in Kenya

**Figure 2:**
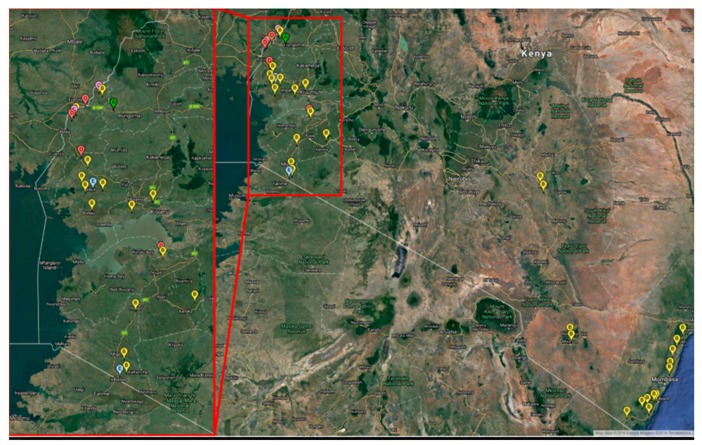
Map of Kenya showing distribution of cassava whiteflies included in this study. The red box highlights the collection sites near Lake Victoria, and the red box to the left is a blow-up of that region. The different coloured circles with letters represent the five different clades *Bemisia tabaci* species (A–E or SSA-A–E) as indicated in Table 1 in the column labelled “Genetic Group”.

**Figure 3:**
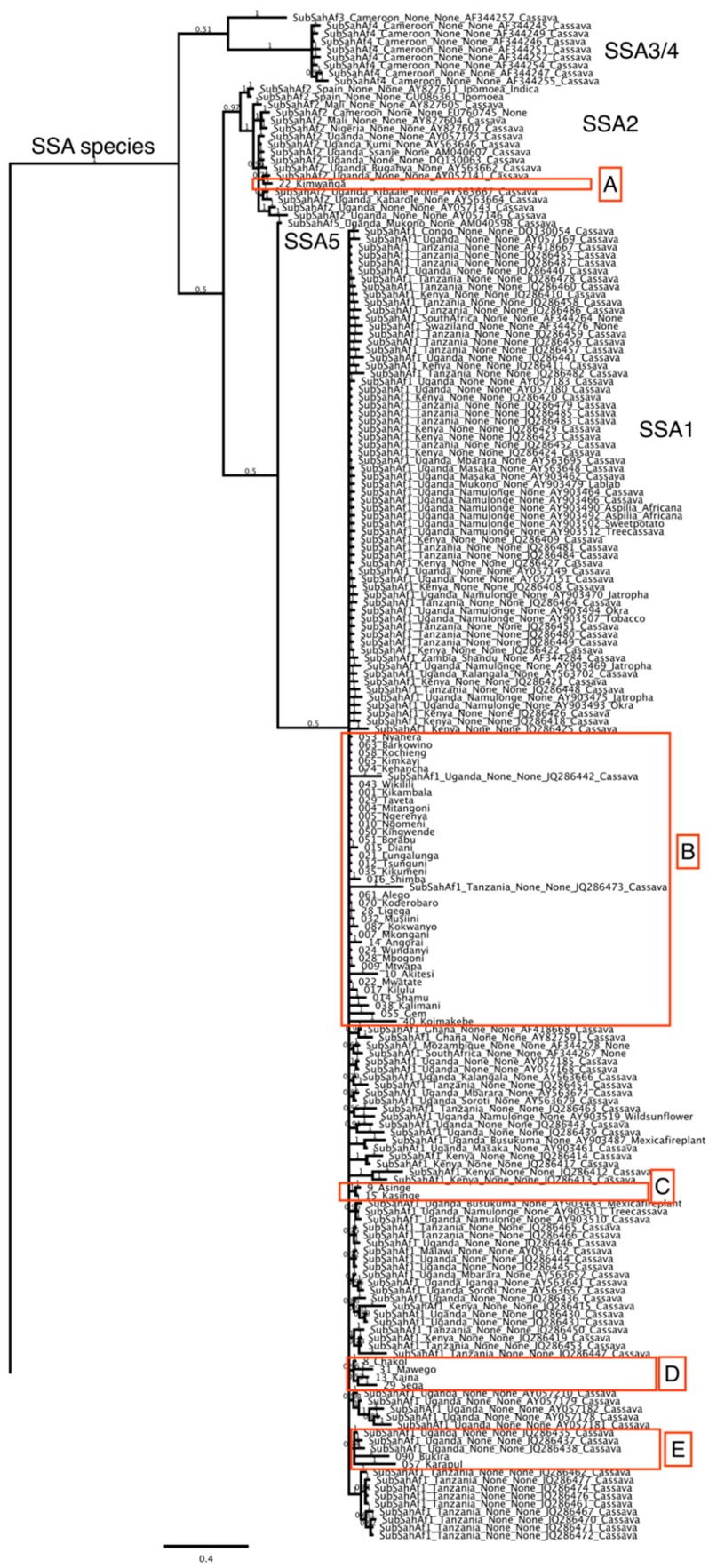
Entire MrBayes phylogenetic tree based on the mitochondrial cytochrome oxidase I sequence for *B. tabaci* collected in Kenya. Figure A1. (a–c) Distinct clades A–E blown up below with new sequences generated in this study highlighted in green.

**Figure 4:**
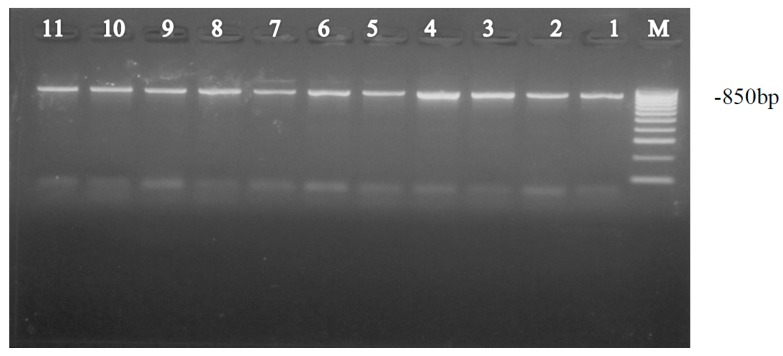
Polymerase chain reaction-amplified product (~850 bp) of *B. tabaci*.

Amplification of the mtCO1 gene resulted in an 850 bp fragment (Figure 4) for each adult whitefly using pair primers MT10 and MT12. A consensus sequence from both forward and reverse sequencing was obtained for the 44 individual whiteflies. The 44 new mtCOI sequences from this study were combined with other mtCOI sequences from global whitefly samples found in www.whiteflybase.org. The final dataset was composed of 659 sequences and was trimmed to 650 bp. The results of the current study indicate the distribution of two different *Bemisia tabaci* sub-Saharan Africa species circulating in Kenya (Figure 3). The putative species [45] found in our study are from the sub-Saharan Africa species SSA1 and SSA2, in which five distinct clades were identified and labelled (clades A–E) (Table 1 and Figure 2). The first clade (A) can be compared to the SSA2 putative species [25] with sequence similarity of 99.5% which was also referred to as the “invader” [28]. According to Legg et al. [28] SSA2 is frequently found in western Kenya and is an invasive vector in cassava growing areas which is associated with increased incidences of CMD in various geographical regions of East Africa [46]. The geographic distribution of the species found in Kenya is intriguing. Only SSA-B was found at the coast while around Lake Victoria there were four different genetic entities (SSA-A, B, C, D) identified (Figure 3 and Table 2). The second clade (B) clustered with other *B. tabaci* sequences that have sequence similarity (97%–98.8%) from throughout east and southern Africa [47] (Figure 3). Thirdly, clade C specimens were collected from one county, Busia, and two samples from the region form a unique clade to Kenya not found before in previous sampling efforts in the region. Fourthly, clade D, sampled from counties surrounding the Lake Victoria Basin and from Busia, is also unique to Kenya. Finally, the last clade (E) clustered with the sub-Saharan Africa (SSA1) based upon considering previously published sequences from southern Uganda [28].

## 4. Discussion

Using the mtCOI gene as a molecular marker, our study determined the occurrence of two *Bemisia tabaci* species belonging to five distinct clades within the sub-Saharan Africa (SSA1 and SSA2) species (Table 2). SSA-B is the most abundant clade found in Kenya and is widely distributed along the coast of Kenya and around the Lake Victoria basin area. This corresponds to where cassava is grown in Kenya [9]. The nature of the agroecological zones where cassava is grown in Kenya is hot and humid [36], which also supports many other crops that become alternative hosts of whiteflies, such as sweet potato and tomato, thereby increasing the spread of the vector and transmission of viral diseases in cassava. Whitefly collections from cassava fields (three to six months) from major cassava growing areas in Kenya have a close relationship with the virus diseases that are widely distributed in different agro-ecological zones (low, medium, and high altitude at less or greater than 1000 m above sea level [14]. The Lake Victoria Basin climate is an equatorial climate with temperatures modified by the relatively high elevation of the mountains surrounding the basin, such as Mt. Elgon. Temperatures and rainfall are lower than the typical equatorial conditions to be classified as a sub-humid climate (temperature range between 20 °C to over 35 °C). The rainfall ranges between 1000 mm and 1500 mm with no distinct dry season in the year, thereby creating a favourable atmosphere for SSA whiteflies to spread throughout the lake basin and riparian states. However, along the coast, the climatic conditions are quite different thereby highlighting the diverse habitat SSA-B can inhabit. CMD and CBSD are both cassava virus pandemics that cause 100% loss to cassava preproduction in most parts of East Africa [46]. There is continued spread of CBSD in most areas of East Africa affected by CMD, probably because of large populations of *B. tabaci* during the early planting seasons when the rains are available [11].

In terms of CBSD, the coast is reported to be the original epidemic zone of CBSD, while the Great Lakes regions are the most recent epidemic zone [48]. One hypothesis is that the native range of SSA-B is along the coast in Kenya and appears to be displacing the local species of Nyanza and the Western regions by invading these new areas. The alternative hypothesis is that SSA-B originated in the Lake zone and has been moved to the coast via the movement of cuttings that could potentially be infested with whitefly nymphs. To test these hypotheses, a thorough population genetics study is needed to detect migration patterns of SSA-B. There are other members of the *B. tabaci* complex that are also widespread and invasive. For example, it has been reported that in many parts of the world there has been an explosive outbreak of *B. tabaci* species in the tropics and sub-tropic regions, where species *B. tabaci* MEAM1 and *B. tabaci* MED are extremely polyphagous [30]. These species have the ability of exhibiting resistance to many insecticides, high fecundity, and the capability to displace their competitors [49]. Through close monitoring of *B. tabaci* MEAM1, it has been observed to cause significant losses through its ability to rapidly expand its population, transmit Geminiviruses, and overcome the effects of insecticides [50]; the same could be true for SSA-B.

**Figure A1:**
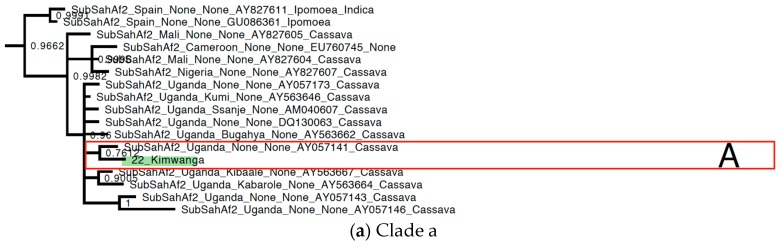

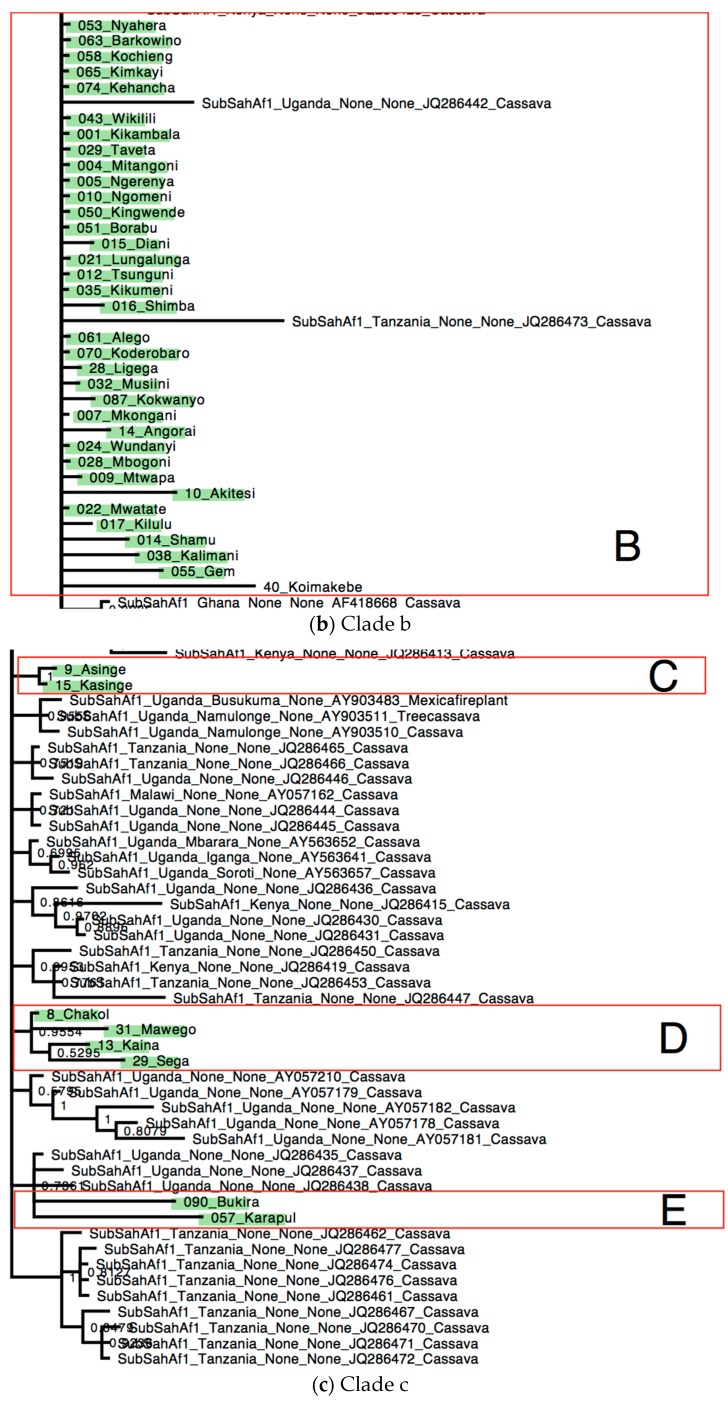
(**a–c**): Distinct clades A–E blown up below with new sequences generated in this study highlighted in green.

In addition, Figure A1c shows that clades C and D of *B. tabaci* are unique and are spread around Lake Victoria Basin of Kenya and Uganda where the cassava crop is highly affected by the CMD and CBSD epidemics. Work done on the epidemiology of both CMD and CBSD in most parts of East Africa is associated with the presence of *B. tabaci* [51], however, most previous studies have failed to correctly characterize the species of *B. tabaci* found. Clade C can only be found in one locality in Busia county away from the Lake Victoria Basin region (Figure 2). The viral diseases are rapidly spread from plant to plant and between fields by the whitefly vectors, *B. tabaci*, and producing the phenomenon of a spreading severe disease “front” that advanced through the southern part of Uganda and to the neighbouring countries [11]. The phylogenetic analysis of the whiteflies in Kenya indicates the occurrence of five different clades of *B. tabaci* from the surveyed areas, which suggests close relationships within the cryptic species complex as well as the evolutionary history within the riparian states of East Africa [52].

In the past, clade A (SSA2) was a major genetic group prevalent in areas affected greatly by the CMD pandemic that most recently has been reported to be absent from cassava collections in Uganda [8,12,31] and in western Kenya [8,44]. This study reports the detection of SSA2 in western Kenya along the boundary of Kenya and Uganda. SSA2 is referred to as an invasive whitefly species “invader/Ug2” that is associated with areas that are severely affected by CMD in Uganda [53,54]. The putative species SSA2 most probably moved from Uganda to Kenya through exchange of infested cassava planting materials and/or environmental influences, such as the effect of strong winds.

There are many viral species and many whitefly species circulating in East Africa. The causal viruses of CMD are: *African cassava mosaic virus-Kenya* (ACMV-K), and *East African cassava mosaic virus* (EACMV), *East African cassava mosaic Zanzibar virus* (EACMZV), *Uganda variant strain of the EACMV* (EACMV-Ug), *and East African cassava mosaic Kenya Virus* (EACMKV) and for cassava brown streak disease there are two recognized species, *Cassava brown streak virus* and *Uganda cassava brown streak virus* with many more being uncovered as more genomes are characterized [14,15]. The challenge now is to match these many viruses of CMD and CBSD with the many species in the *Bemisia tabaci* species complex. We can no longer assume the vector is “*B. tabaci*” as there are many species present in the region where these devastating viruses are circulating. All studies in the future must include barcoding of the vector they are finding in their survey data and the transmission studies.

## 5. Conclusions

In sub-Saharan Africa, cassava remains primarily a subsistence crop to farmers, but little attention has been directed to knowledge and significance on whitefly management on cassava, and this has led to high populations and continued spread of the vector and transmission of the virus diseases in cassava crops [11]. The notorious plant virus vector *B. tabaci* in Kenya is made of two species (SSA1 and SSA2) grouped into five distinct clades. The five clades have a less than 3.5% divergence in mtCOI [25], but they may differ in terms of their biology, fecundity, virus transmission, and mating ability [8], and, as such, further biological studies are needed. In addition, the SSA2 putative species requires further investigation of its role in cassava virus disease epidemiology.

## Figures and Tables

**Table 1:** Summary *B. tabaci* collected in Kenya and used in this study. These data are also mapped onto Figure 2, and there is an interactive map found at http://beta.whiteflybase.org/datamap/.

Sample Number	County	Area	Field	Geographic Location	Altitude	Genetic Group	Accession No.
1	Siaya	Ligega	NF3	N00.20321, E034.27061	1330	SSA-B	KY523872
2	Siaya	Sega	NF4	N00.27314, E034.22346	1243	SSA-D	KY523885
3	Siaya	Gem	NF5	N00.03174, E034.25409	1957	SSA-B	KY523894
4	Siaya	Karapul	NF8	N00.05285, E034.30654	3132	SSA-E	KY523892
5	Kisumu	Nyahera	NF2	S00.03051, E034.71960	1459	SSA-B	KY523852
6	Siaya	Kochieng	NF10	N00.09266, E034.23024	1256	SSA-B	KY523854
7	Siaya	Alego	NF14	N00.04531, E034.37395	1409	SSA-B	KY523870
8	Kisumu	Kit mikayi	NF22	S00.10358, E034.57415	1176	SSA-B	KY523855
9	Siaya	Barwino	NF16	S00.09508, E034.29937	1509	SSA-B	KY523853
10	Homabay	Koderobaro	NF33	S00.78241, E034.59921	1416	SSA-B	KY523871
11	Migori	Bukira West	NF34	S01.23.442, E034.49201	2363	SSA-E	KY523887
12	Homabay	Kokwanyo	NF27	S00.42202, E034.78882	1275	SSA-B	KY523875
13	Busia	Chakol	WF12	N00.52575, E034.16062	1152	SSA-D	KY523877
14	Busia	Asing’e	WF13	N00.54886, E034.17672	1152	SSA-C	KY523880
15	Busia	Aktesi	WF14	N00.56531, E034.18919	1153	SSA-B	KY523886
16	Busia	Kaina	WF17	N00.62388, E034.25398	1177	SSA-D	KY523876
17	Busia	Ang’orai	WF18	N00.69299, E034.37015	1230	SSA-B	KY523879
18	Busia	Kasing’e	WF19	N00.71344, E03434693	1393	SSA-C	KY523881
19	Bungoma	Kimwanga	WF26	N00.59644, E034.44701	1233	SSA-A	KY523874
20	Taita Taveta	Wundanyi	CF30	S03.39443, E038.36485	1339	SSA-B	KY523882
21	Homabay	Mawego	NF31	S0038946, E034.77587	1424	SSA-D	KY523888
22	Migori	Kiomakebe	NF40	S01.21158, E034.53714	1562	SSA-B	KY523895
23	Migori	Kehancha	NF41	S01.11733, E034.52141	1418	SSA-B	KY523856
24	Nyamira	Borabu	NF44	S00.72273, E035.00735	1926	SSA-B	KY523864
25	Kilifi	Kikambala	CF1	S03.86207, E039.74467	86	SSA-B	KY523858
26	Kilifi	Mitangoni	CF5	S03.69020, E039.77895	61	SSA-B	KY523860
27	Kilifi	Ngerenya	CF7	S03.54610, E039.83553	53	SSA-B	KY523861
28	Kilifi	Mkongani	CF9	S03.39686, E039.91546	20	SSA-B	KY523878
29	Kilifi	Mtwapa(KARLO)	CF12	S03.93400, E039.73534	38	SSA-B	KY523884
30	Kwale	Ngombeni	CF13	S04.12535, E039.63130	25	SSA-B	KY523862
31	Kwale	Tsunguni	CF15	S04.18433, E039.55974	115	SSA-B	KY523867
32	Kwale	Shamu	CF17	S04.30154, E039.54972	42	SSA-B	KY523891
33	Kwale	Diani	CF18	S04.30731, E039.51998	90	SSA-B	KY523865
34	Kwale	Shimba hills	CF19	S04.35600, E039.42932	104	SSA-B	KY523869
35	Kwale	Kilulu	CF21	S04.39463, E039.35639	85	SSA-B	KY523890
36	Kwale	Kikoneni	CF22	S04.42855, E039.31791	70	SSA-B	KY523868
37	Kwale	Lungalunga	CF25	S04.53264, E039.14732	64	SSA-B	KY523866
38	Kwale	Kingwende	CF27	S04.48550, E039.45035	22	SSA-B	KY523863
39	Taita Taveta	Mwatate	CF28	S03.48400, E038.38088	830	SSA-B	KY523889
40	Taita Taveta	Mbogoni	CF36	S03.41414, E037.70263	729	SSA-B	KY523883
41	Taita Taveta	Taveta	CF37	S03.46017, E037.69030	720	SSA-B	KY523859
42	Kitui	Kalimani	EF18	S01.31211, E037.95479	1232	SSA-B	KY523893
43	Kitui	Wakulili	EF25	S01.42728, E037.99684	1135	SSA-B	KY523857
44	Machakos	Musiini	EF4	S01.42061, E037.36724	1413	SSA-B	KY523873

**Table 2:** Geographic distribution of the five distinct clades of whiteflies found in Kenya. SSA-A-E corresponds to the clades found in Figure 3.

Location in Kenya	Number of Samples	SSA-A	SSA-B	SSA-C	SSA-D	SSA-E
Western/Lake	7	1	2	2	2	-
Nyanza	16	-	12	-	2	2
Eastern	3	-	3	-	-	-
Coast	18	-	18	-	-	-
